# Transition From Observational to Collaborative Learning to Augment Practical Skill Training in First-Year Medical Students

**DOI:** 10.7759/cureus.41899

**Published:** 2023-07-14

**Authors:** Madhusudhan Umesh, Vidya Singaravelu, Kalpana M, Archana Gaur, Vidya Ganji, Madhuri Taranikanti, Nitin John, Sai Saileshkumar

**Affiliations:** 1 Physiology, All India Institute of Medical Sciences, Bibinagar, Hyderabad, IND; 2 Pediatrics, Malla Reddy Institute of Medical Sciences, Hyderabad, IND; 3 Physiology, NRI Institute of Medical Sciences, Vizag, IND

**Keywords:** situated learning, group learning, skill training, practical demonstration, collaborative learning

## Abstract

Introduction

Students exhibit less interest in hematology demonstration experiments as they are not expected to do it during their examination. Adopting a different strategy, like collaborative learning, might spark interest, motivate them to work together towards a shared objective, and help further learning and understanding. The current study aimed to assess the effectiveness of collaborative learning in comparison with traditional practical demonstration.

Methodology

First MBBS students were divided into two groups of 50 each (1 - collaborative learning, 2 - traditional demonstration). In the traditional demonstration, the experiment was demonstrated by faculty using the required materials. In the collaborative learning method, 50 students were divided into groups (seven of seven each) and each group was provided with procedural details of the experiment and requisite materials. At the end of the experiment, assessment was done. In collaborative learning groups, the team cohesion scale (TCS) was employed to analyze group dynamics. Students’ perceptions, and feedback regarding collaborative learning as a tool in practical experiments were collected using a five-point Likert scale.

Results

Post-experiment assessment scores in collaborative learning (8.65±1.54) were significantly higher than the traditional demonstration group (7.06±1.46). High scorers in TCS consistently belonged to groups that completed the experiment on time (positive outcome), whereas students with low scores often belonged to groups that did not complete the experiment (negative outcome).

Conclusion

Collaborative learning may be used for practical teaching in medical education as it fosters good communication, enables problem-solving, aiding the Indian medical graduate in fulfilling the role of a team member.

## Introduction

Collaborative learning and cooperation are essential skills that influence the quality of health treatment [1]. Worldwide, medical schools are being advised to emphasize the development of these abilities from an early age [2]. Medical students frequently collaborate and study together in small group learning situations at the outset of a medicine program, emphasizing the value of collaborative learning [3].

Collaborative learning is described generically as "students collaborating in groups of two or more, looking cooperatively for answers, or meanings, or accomplishing a task or generating a product [3]. Collaborative learning activities vary greatly, but the majority focus on the students' investigation or application of the course information, rather than the teacher's presentation or explanation of its content [4].

Collaborative learning of topic knowledge is a prevalent notion in health professional education, and it takes place in several small-group learning formats, such as problem-based learning (PBL) and team-based learning (TBL) [5].

To work as a group, you need good cohesion with the group members. Cohesion is a dynamic process, which is reflected in the tendency for a group to stick together and remain united in the pursuit of its goals and objectives [6], also described in expressions such as solidarity, team spirit, and morale [7]. At the end of the day, the results or performance of the group depends on communication, behavior, mutual understanding, loyalty, and commitment among the team members.

Students typically exhibit less interest in normal Hematology demonstration experiments such as absolute eosinophil count, reticulocyte count, platelet count, etc., since they are not expected to do it individually during their final examination. So, adopting a different strategy, such as collaborative learning, might spark interest in the experiments, motivate them to work together towards a shared objective, and help further learning and understanding.

With this background, the current study was planned with the objectives to assess the effectiveness of collaborative learning in comparison with traditional practical demonstration, to determine the group dynamics using the team cohesion scale, and to assess the student perception of collaborative learning using the five-point Likert Scale.

## Materials and methods

The study was conducted after obtaining IRC from Little Flower Hospital & Research Centre (EC/12-26) & Institutional Ethical Committee clearance. Students were informed about the method of conduction & informed written consent was obtained in the prescribed format.

Study population, setup, and sample size

The study population consisted of first-year MBBS students at DM Wayanad Institute of Medical Sciences (DMWIMS). The study was setup in the hematology lab and demonstration room of the physiology department. The sample size of the study was 100. 

Procedure of collaborative learning

First-year MBBS students in batch A comprising 50 students were included in this mode of learning. They were divided into seven groups consisting of seven members in each group. The students were grouped based on their performance in the first internal examination (mid-semester exam). Each group included:

· high performers (who scored more than 80% in mid-semester exams),

· average performers (who scored between 50-80% in mid-semester exams) and,

· low performers (who scored less than 50% in mid-semester exams).

All groups were provided with a copy of the procedure to be followed while performing the experiment with all necessary apparatus & reagents. Each group was monitored by separate faculty members. Each group has to perform the task and complete the experiment within the stipulated time and show the results to the faculty assigned to them.

At the end of the experiment, the assigned faculty conducted an assessment that included standardized questions that were practical-oriented.

At the end of the session the students were administered a self-assessment tool, the team cohesion scale (TCS), which assessed the group dynamics. Students' perception and feedback regarding collaborative learning as a tool in practical experiments was then collected using a five-point Likert scale.

Team cohesion scale (TCS) 

This scale is a self-assessment tool where participants use a five-point scale to assess the importance and utility of each statement. Participants indicate their responses by choosing any one of the five options (1 = least agreement, 2 = a little agreement, 3 = neutral, 4 = much agreement, 5 = most agreement). The questionnaire made of 19 items, explored three main factors - goal orientation, open communication, and mutual understanding.

Goal Orientation

This section aimed to explore a set of behaviors taken to attain specified goals, which may be both a construct of scholastic achievement and a willingness to innovate in achievement settings. It consisted of eight items that covered the topics of goal commitment, team performance, team communications, attachment to the team, positive attitudes to the goal, discussion of errors, contributions to each other, and members’ enthusiasm. Goal orientation has the potential to forecast team processes and outcomes and its existence motivates and governs the team's behavior.

Open Communication

Communication is the sharing of information and behaviors between persons and encompasses written, oral, nonverbal, and technological means. In high-cohesion teams, gossip as open communication between members will enhance the flow of information and foster loyalty. This section was made up of eight components that symbolize creating awareness, requesting recommendations, work norms and interactions, rapid replies, clarity and consensus, belongingness, decision-making, and team members' contributions to the team objective.

Mutual Understanding

Mutual understanding is generated by cooperative or helpful behavior, but it cannot occur without agreement, empathy, amity, trust, agreement, unity, goodwill, and harmony among members. Thus, trust fosters collaboration, commitment, and the building of collective identities in organizations. This section included three items - representing task reviews, work values, and prevention of errors.

Likert Scale

The students’ perceptions regarding the collaborative learning exercise were collected using the five-point Likert scale with the following items - If the method was intriguing whether it was simple to implement, whether it aided comprehension, whether they believe it would improve their performance, and whether they believe it would aid in better retention of the subject, with students evaluating it from 1 to 5, strongly disagree to strongly agree.

Procedure for practical demonstration

First-year MBBS students in batch B comprising 50 students were selected for this mode of learning. A practical experiment was demonstrated by the faculty in the traditional manner using the required apparatus and reagents, after which an assessment was conducted with the same questions used in the collaborative learning group. After this, there was a cross-over of the groups to eliminate ethical conflicts.

Statistical Test

The data was entered in MS Excel (Microsoft Corporation 2019). and SPSS (IBM Corp. Released 2013. IBM SPSS Statistics for Windows, Version 22.0. Armonk, NY: IBM Corp) was used for the analysis of data. The outcome variables were summarized using mean, standard deviation (SD), and percentage. The mean of test scores between the two groups was compared using a t-test. p-value < 0.05 was considered statistically significant.

## Results

**Figure 1:**
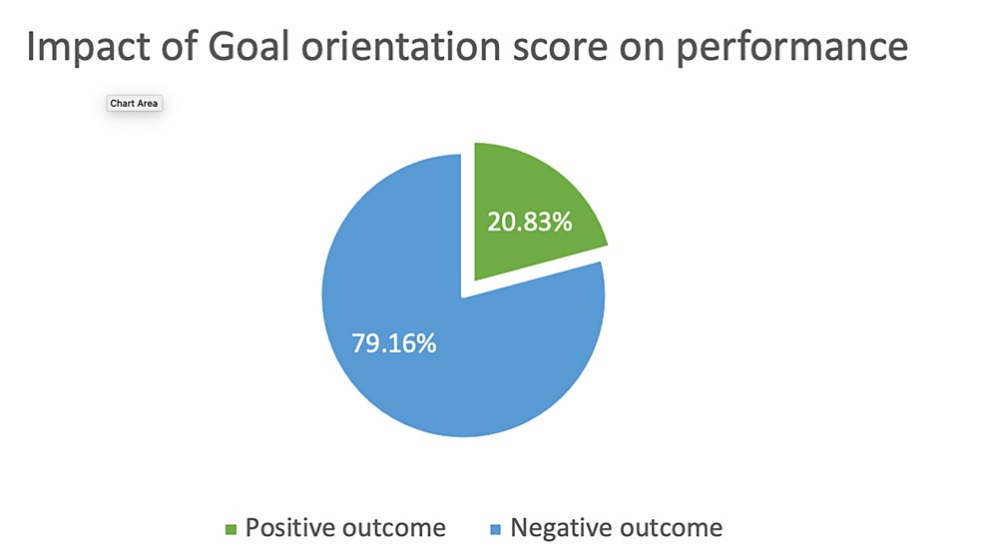
Impact of Goal orientation score on performance Out of 24 students who had scored less than normal in the goal orientation component majority 79.16% (19) belong to those two groups who were not able to complete the experiment within the given time (negative outcome).

**Figure 2:**
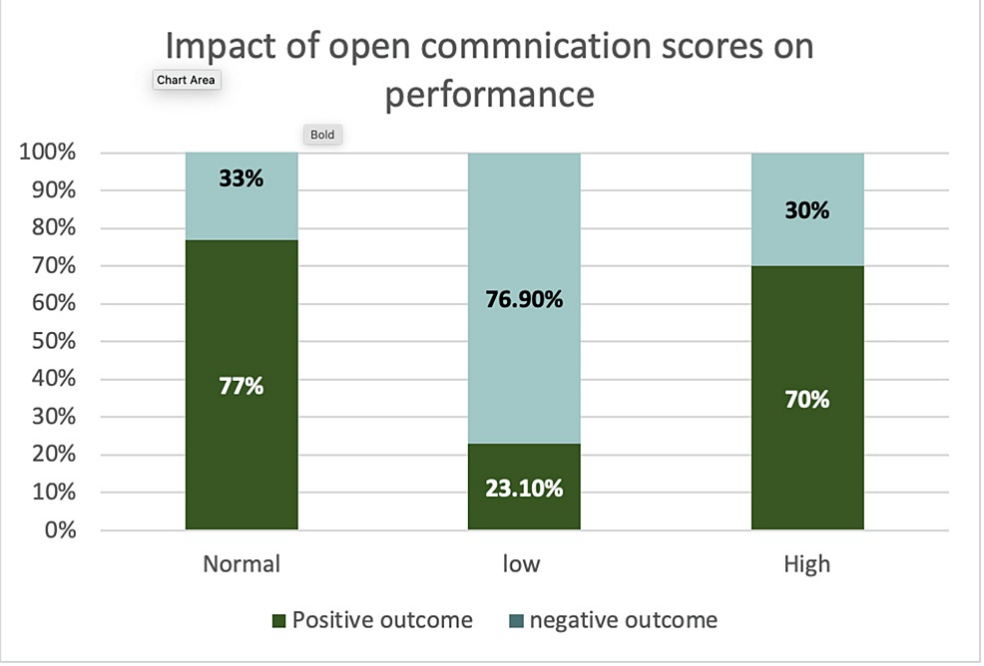
Impact of open communication scores on performance Students with normal or higher open communication scores consistently belonged to the groups that completed the experiment in the allotted time (positive outcome), whereas students with low scores most often belonged to the groups that did not complete the experiment (negative outcome).

**Figure 3:**
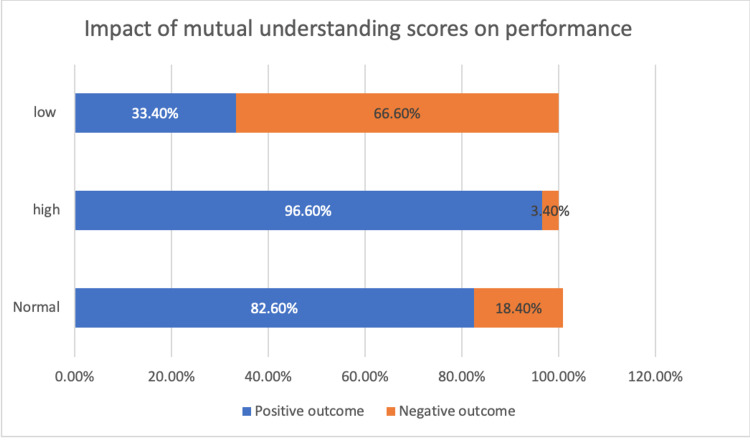
Impact of Mutual understanding scores on performance Students with normal or higher open communication scores consistently belonged to the groups that completed the experiment in the allotted time (positive outcome), whereas students with low scores most often belonged to the groups that did not complete the experiment (negative outcome).

Out of 100 first-year MBBS students, 50 were subjected to collaborative learning and 50 to the traditional demonstration method. In the collaborative learning group, 56% (28) were females whereas 44% (22) were males. In the traditional demonstration group, 46% (23) were females and 54% (27) were males. The mean age of students in the collaborative learning group was 17.2±2.34 and in the traditional demonstration group was 17.5±2.58 which was not significantly different in both the groups. The assessment was conducted for 10 marks and the scores at the end of the exercise in both groups are tabulated in Table 1. It can be seen that the collaborative group scored significantly better than the traditional demonstration group. Out of seven groups, five groups were able to focus the cell, identify & could complete the experiment successfully within the stipulated time, whereas the other two groups were not able to do so. To analyze the group dynamics team cohesion scores were used, the scores are represented in Table 2, & its impact on students’ performance is shown in Figures 1, 2, 3. Student's feedback shown in Figure 1, reflects that the collaborative learning method is interesting, engaging, easy to understand & helps in better retention of the concept than compared to the traditional demonstration group.

**Table 1:** Assessment scores comparison in both the groups *Statistically significant

Groups	Test scores	t value	P value
Collaborative learning group	8.65±1.54	5.3	0.001*
Traditional demonstration group	7.06±1.46		

**Table 2:** Team cohesion scores analysis

Components	Score level		
	Normal (29-38)	Low <28	High >39
Goal orientation	52% (26)	48% (24)	NIL
	Normal (30-38)	Low <29	High >39
Open communication	48% (24)	26% (13)	26% (13)
	Normal (10-14)	Low <9	High >15
Mutual understanding	52% (26)	18% (9)	30% (15)

## Discussion

According to the findings of this study, collaborative learning is not only a viable and effective technique to understand study material, but it is also a tool for developing communication skills, team building, and leadership traits. 

In group learning, members are likely to speak with one another and come up with an acceptable solution to an issue. The introduction of team/group work at an earlier stage of medical education has resulted in a stronger impact [8]. With the implementation of competency-based medical education (CBME), teaching has evolved towards student-centered learning, problem-based learning, and community-based education, with numerous innovative teaching-learning approaches being investigated to increase student-centered learning.

Quite a number of studies have proven that collaborative learning enhances communication skills, problem-solving abilities & newer ways of approaching a complex problem [9-12]. We analyzed group dynamics using the team cohesiveness scale, which is commonly used in sports that require teamwork to succeed, and it has been established that the stronger the cohesion in the group, the greater the likelihood of victory [13]. This scale can assess team cohesion in three crucial areas - goal orientation, open communication, and mutual understanding/respect. Those with high cohesion completed their tasks within the allocated time in this study, whereas those with low cohesion did not. As illustrated in Figures 1, 2, 3, Individuals who were able to easily integrate into the group, provide valuable suggestions, listen to and appreciate the opinions of other team members, and wrapped up the task effectively got high cohesion scores.

First-year medical students are often enthusiastic learners, but once they realize that a specific experiment will not be tested in their examination, they lose interest and put forth only the bare minimum of effort to understand the essential principles of the experiment. This makes the practical demonstration sessions less exciting and invites monotony. To make these sessions more interactive, we employed the collaborative learning method, as illustrated in Figure 4. The majority of students (80%) thought it was superior to the usual method of demonstration.

According to the National Medical Commission (NMC) undergraduate (UG) curriculum, ESR (erythrocyte sedimentation rate), PCV (Packed cell volume), and osmotic fragility must only be demonstrated in hematology practicals of first-year MBBS [14]. In some universities, studies such as estimating platelet count, reticulocyte count, and absolute eosinophil count are only available to postgraduate students or are only available for demonstration. Students sometimes underestimate the significance of these experiments since they are not examined in summative exams. So, we established this form of a teaching-learning tool in which students try to get oriented to their purpose and operate as a team by communicating effectively and displaying mutual respect among team members, finally leading to successful task completion.

Limitations

Although the study solely addresses collaborative learning in hematology practicals, the overall outcome of group learning cannot be evaluated. If alternative practical exercises, such as problem-based scenarios, could have encouraged newer/innovative problem-solving concepts.

## Conclusions

Since group learning requires members to communicate with one another and come up with a suitable solution to a problem, we may use group learning for various practical/clinical skills in medical education. It will also help foster the attitude required to be a team member thus fulfilling one of the roles of the Indian Medical Graduate.
